# Conflicting Interests in the Pathogen–Host Tug of War: Fungal Micronutrient Scavenging Versus Mammalian Nutritional Immunity

**DOI:** 10.1371/journal.ppat.1003910

**Published:** 2014-03-13

**Authors:** Joanna Potrykus, Elizabeth R. Ballou, Delma S. Childers, Alistair J. P. Brown

**Affiliations:** Aberdeen Fungal Group, School of Medical Sciences, Institute of Medical Sciences, University of Aberdeen, Foresterhill, Aberdeen, United Kingdom; Duke University Medical Center, United States of America

## Introduction

Strife concerning the accessibility of essential trace elements, such as transition metals, represents an important aspect of the dynamic interaction between a pathogenic fungus and its mammalian host. The host defends itself against infection by sequestering these essential micronutrients away from the invading pathogen via a phenomenon termed “nutritional immunity” [1]. In turn, the fungus employs an array of tactics (scavenging and storage) to hoard micronutrients and support growth when these resources are scarce. In addition, micronutrient limitation triggers the expression of virulence determinants that can aggravate disease [2]–[4].

Half of all enzymes and a third of all proteins require metals for their functionality [5], yet many transition metals are insoluble and potentially toxic at neutral pH and in an oxygenated, aqueous milieu. For example, iron and copper are redox active and capable of generating free radicals via Fenton chemistry under these conditions [6]. Furthermore, toxic excess of a non-native metal can displace the native metals from metalloproteins or inhibit the function of non-metalloproteins. Therefore, the levels of iron, copper, and zinc and their partitioning within the body must be tightly regulated to maintain cellular homeostasis whilst avoiding cellular damage.

In the mammalian host, iron is the most abundant transition metal (3–4 g/adult human), followed by zinc (1.4–2.3 g/adult human), with copper trailing behind (∼100 mg/adult human) [7]. These metals have roles in signalling, immune responses, physiology, and development [6], and both metal deficiency and overload are detrimental to the host. Iron deficiency, the most common dietary deficiency in the world, weakens the immune system, whilst copper deficiency renders the host more susceptible to infection [5], [8]. On the other hand, iron overload is a significant risk factor for some fungal diseases, especially for mucormycosis [9]. Furthermore, individuals with acute myeloid leukemia, characterised by increased serum iron, have a higher risk of *Candida* and *Aspergillus* infections [10]. Iron overload also exacerbates meningoencephalitis in a mouse model of cryptococcosis [11], [12]. Conversely, fungal pathogens depend on effective metal acquisition mechanisms for full virulence [3], [13]–[16].

## The Many Faces of Nutritional Immunity

The mammalian host exploits both “constitutive” and “inducible” nutritional immunity to sequester transition metals away from microbial pathogens. The host regulates its interchangeable metal pools by sequestering metals via protein carriers or by partitioning them between intracellular stores [1], [9]. This essentially imposes constitutive nutritional immunity by depleting the extracellular milieu of essential metals and creating a micronutrient-limiting environment for the pathogen. For example, the estimated serum concentration of free ferric iron is 10^−24^ M, many orders of magnitude lower than that expected based on its solubility (10^−9^ M) [17]. Likewise, the intracellular “free pool” of copper is less than one copper atom per cell [18]. Pathological disruption of this constitutively low metal ion environment is one factor that predisposes patients to fungal infection [10], [18].

Upon infection, the host imposes “inducible” nutritional immunity by readjusting its global micronutrient homeostasis to further limit microbial access to endogenous metal ion pools. This response is achieved both on systemic and local levels. Systemic metal readjustments are observed, such as the hypoferraemia associated with disseminated infections. In this case, regulation is achieved via the secreted hepatic hormone hepcidin, which itself is responsive to various regulators, such as IL-6 [19]. Indeed, disseminated *C. albicans* infections are accompanied by increases in hepcidin and decreases in the degree of iron saturation of serum transferrin [20]. At the local level, inducible mechanisms include the secretion by macrophages and neutrophils of siderocalins (such as lipocalin-2), which bind siderophores [1] (vide infra). The neutrophil copper-binding protein calgranulin C might also contribute to nutritional immunity [21].

Most data on nutritional immunity relate to bacterial infections. However, there is evidence for localised, induced nutritional immunity during systemic fungal disease. During disseminated candidiasis, infiltrating neutrophils appear to seal off the developing fungal lesions from the rest of the tissue to create a metal-deprived environment [22]. Macrophages parasitized by *Histoplasma capsulatum* are reprogrammed to exclude zinc and, probably, iron [23]. Neutrophil extracellular traps (NETs), which can control fungal growth via either cidal (*C. albicans*, *Cryptococcus neoformans*) or static mechanisms (*Aspergillus fumigatus*), are decorated with proteins such as the zinc-scavenging calprotectin [24]. Furthermore, the release of the polymorphonuclear leukocyte protein lactoferrin at sites of infection inhibits the growth of *C. albicans* and *A. fumigatus*, presumably by inducing localized iron deprivation [25].

Metal deprivation is not the only means by which the host manipulates micronutrient levels to contain an infection. A contrasting mechanism, involving the “poisoning” of microbes with excess metal ions, is gaining attention in the literature [16]. An elegant example is the case of copper poisoning during *Cr. neoformans* infection, where copper redistribution in alveolar macrophages is countered by expression of fungal metallothioneins sequestering the toxic excess of metal [16].

## Overcoming Host Nutritional Defences—Many Tricks up the Fungal Sleeve

Fungi exploit many mechanisms to scavenge metal ions from different substrates, adjusting their metal acquisition machinery as required [26]. The molecular mechanics that underlie metal sensing remain unclear [5], and the regulatory networks that control metal acquisition systems are complex, responding not just to micronutrient availability but also to environmental cues such as carbon source, hypoxia, and pH. These are reviewed elsewhere (e.g., [2], [3], [14]). Here, we summarise the fungal micronutrient acquisition pathways themselves and discuss how they respond to nutritional immunity.

### Multiple acquisition pathways

Low and high affinity fungal transporters have been characterised for copper and zinc, but little is known about the acquisition of these metals in vivo. Notable exceptions include the Pra1/Zrt1-based zinc uptake system in *C. albicans* [27] and copper acquisition by *Cr. neoformans* via *CTR1* and *CTR4* [16], [28]. In contrast, iron acquisition is relatively well researched.

**Figure 1:**
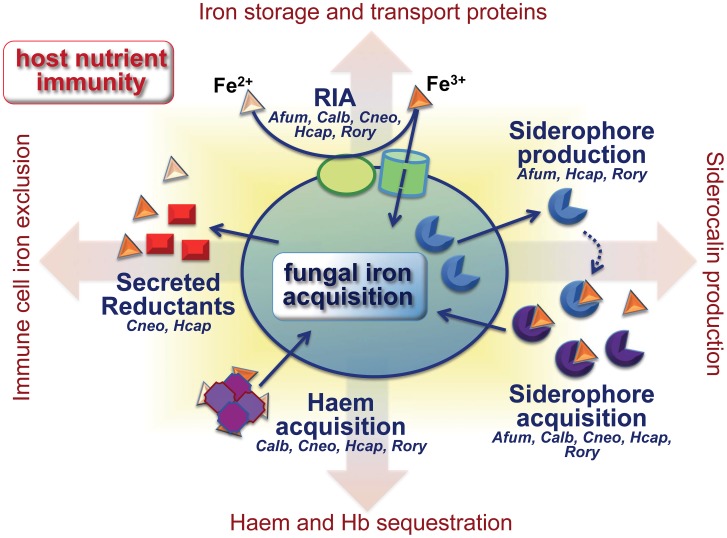
Fungal iron scavenging competes with mammalian nutritional immunity. Pathogenic fungi employ different combinations of evolutionarily related strategies to acquire iron in the limiting microenvironments of the host. The figure focuses on those fungal species for which data are currently available. See text for details. Hb: haemoglobin, RIA: reductive iron acquisition, *Afum*: *A. fumigatus*, *Calb*: *C. albicans*, *Cneo*: *Cr. neoformans*, *Hcap*: *H. capsulatum*, *Rory*: *R. oryzae*.

Complex iron homeostasis within the host involves a plethora of storage proteins (e.g., ferritin), carrier proteins (e.g., transferrin, lactoferrin), and the machinery for senescent red blood cell recycling [6]. The continual transfer of iron between various body compartments provides potential targets for fungal interference. To access iron, fungi employ: (1) a reductive iron acquisition (RIA) pathway, comprising a permease and a multicopper oxidase for high-affinity ferric import; (2) siderophore transporters for endogenous or exogenous siderophores; (3) secreted and cell surface reductants; and (4) the endocytic pathway for haem internalisation, in conjunction with intracellular haem oxygenase-1 (Hmx1) for haem iron extraction (Figure 1). Not all of these pathways are present in all fungi. For example, *A. fumigatus* mainly exploits endogenous siderophores for iron scavenging and also possesses the RIA system (FtrA/FetC), but only the former pathway is required for virulence [29]. *C. albicans* utilizes RIA (Ftr1/Fet3 and additional Ftr1/ferroxidase-homologue combinations), xenosiderophore transport (Sit1 transporter), and haem acquisition (Rbt5 and other receptors) [30]. *Cr. neoformans* relies on RIA (Cft1/Cfo1), a xenosiderophore transporter (Sit1), secreted and extracellular reductants (3-hydroxyanthranilic acid, melanin), and haem acquisition (Cig1) [11]. *H. capsulatum* employs RIA (Ftr1/Fet3), produces and internalises siderophores, secretes gamma-glutamyltransferase and glutathione-dependent ferric reductase, and utilizes haem (Figure 1) [15], [21].

Certain substrates require specialised surface receptors. For example, *C. albicans* uses the protein Als3 for ferritin iron and the Rbt5 receptor (and other CFEM-type receptors) for haemoglobin and haem [30]. Als3 also acts as an adhesin and invasin. It is expressed during hyphal development, promoting the adhesion and host endocytosis of *C. albicans* cells. This suggests interesting interconnections between micronutrient availability and acquisition and other types of fungus–host interaction during disease progression. Haem is also readily utilised by *Cr. neoformans* via an uptake pathway that involves the secreted haemophore Cig1 [31]. In *Rhizopus oryzae*, the ferric reductase Ftr1 has been implicated in haem iron acquisition [32]. No specific uptake systems for haem, ferritin, or transferrin have been described for *A. fumigatus*, although, like *H. capsulatum*, this fungus uses secreted siderophores to chelate ferric iron from transferrin [15], [33].

The various iron acquisition mechanisms seem to become prominent at different stages of infection. Accordingly, *C. albicans* requires Sit1 for epithelial escape, RIA for the establishment of disseminated bloodstream infections, and Hmx1 for sustaining deep-seated organ infections [13], [30]. In contrast, *H. capsulatum* requires RIA during early infection stages and siderophore biosynthesis later in the infection process [34].

### Scavenging

Siderophores are small organic molecules with ferric iron binding affinities of ∼10^30^ M at neutral pH. Other than the carboxylate siderophore rhizoferrin found in zygomycetes (*R. oryzae*), all other known fungal siderophores are hydroxamates [21]. In *H. capsulatum* and *A. fumigatus*, the *SID1*/*SIDA* gene is required for the first dedicated step in siderophore biosynthesis. This gene plays a role in the later stages of *H. capsulatum* infection and is required for *A. fumigatus* virulence [15], [29], [34]. Furthermore, *A. fumigatus* produces secreted siderophores, as well as intracellular siderophores for hyphal and conidial iron storage [14]. Although *C. albicans* and *Cr. neoformans* do not produce their own siderophores, they can exploit hydroxamate-type xenosiderophores produced by other microorganisms [11], [30].

It is important to emphasise that iron is not the only transition metal limiting to the fungus within the host environment. This is evident from the discovery that fungi also take advantage of induced secretory mechanisms to obtain non-ferric metal ions. For example, *C. albicans* possesses a dedicated zinc scavenging system, comprised of the secreted Pra1 “zincophore” and the Zrt1 transporter [27]. Additionally, methanobactin-like compounds, i.e., secreted scavengers of copper, have been discovered in *Saccharomyces cerevisiae* [35], [36]. However, the prevalence of such molecules among fungal pathogens of mammals remains unknown.

### Storage and conservation

In fungi, the intracellular storage of transition metals is not limited to vacuoles [7]. *A. fumigatus* produces intracellular siderophores for hyphal and conidial iron storage, and both intra- and extracellular siderophores are required for full virulence [14]. Ferritin-like genes have been identified in *A. fumigatus* and *R. oryzae*, but not in basidiomycetous genomes [26]. Copper hyperaccumulation by *Cr. neoformans* suggests the existence of copper storage mechanisms in this pathogen, which might promote fungal survival inside the macrophages (see above) [16], [37].

When micronutrients are scarce, some fungi fine-tune their metabolism towards micronutrient conservation. For example, *Cr. gattii* and *C. albicans* respond to zinc restriction by down-regulating zinc-dependent alcohol dehydrogenases to increase zinc availability for other metalloproteins [3], [38]. Similarly, in *A. fumigatus*, *C. albicans*, and *Cr. neoformans*, iron-conserving mechanisms operate via the transcription factor HapX to down-regulate genes in iron-consuming pathways, such as amino acid metabolism, tricarboxylic acid cycle, respiration, and haem biosynthesis [26].

Autophagy, a major pathway for the bulk degradation of cytosolic components during nutrient starvation, might represent a non-conventional mechanism for recycling and conserving essential micronutrients to sustain hyphal growth in *A. fumigatus*, although its role in vivo is unverified. Other fungi, such as *C. albicans*, also undergo autophagy, but unlike in *A. fumigatus*, this phenomenon is not suppressed by zinc supplementation [39].

### Impact on virulence factors

As already stated, low metal concentrations induce the expression of fungal metal acquisition genes, which themselves are indispensable for host colonisation, dissemination, and virulence for many fungal pathogens [3], [4], [13]–[16]. Furthermore, pathogenic fungi can activate other virulence determinants in response to micronutrient limitation. For example, iron depletion induces capsule formation in *Cryptococci*, which allows the pathogens to escape recognition by the immune system [4], [40]. As another example, iron deprivation of *A. fumigatus* results in the expression of the major surface allergen ribotoxin AspF1, which could potentiate allergic bronchopulmonary aspergillosis [2]. Thus, in the act of depriving fungal pathogens of nutrients to limit their growth, the host can trigger virulence factors that may exacerbate fungal disease.

## Perspectives

The metal tug of war between pathogen and host is a complex and dynamic process, with each party striving to procure and retain essential micronutrients. The great complexity of this interaction is only now being realised. It is becoming apparent that the host responds to infection by redirecting micronutrients via niche- and perhaps infection-stage–specific mechanisms to various ends, e.g., micronutrient limitation versus micronutrient poisoning. On the other hand, the apparent redundancy of some fungal metal acquisition mechanisms needs to be explored, and their essentiality to the different stages of pathogenesis remains to be established. Also, before we can start drawing global comparisons of metal homeostasis in fungal and bacterial infectious agents, more information is required about micronutrient warfare in fungal pathogens outside the small handful of established model organisms. The ultimate challenge will be to integrate the responses of host and pathogen into a holistic model that describes how the host modulates micronutrient homeostasis during infection and how the pathogen responds to these changes, spatially and temporally. New technologies, such as live-animal imaging of fungal gene expression [16], MALDI (matrix-assisted laser desorption/ionization) mass spectrometry imaging of proteins, and 2-D element mapping directly from biological specimens [22], are beginning to illuminate the pathogen–host tug-of-war over micronutrients. We are confident that the future holds many exciting discoveries in this field.
